# Kidney Biopsy in Patients With Monoclonal Gammopathy: A Multicenter Retrospective Cohort Study

**DOI:** 10.3389/fmed.2021.687149

**Published:** 2021-05-24

**Authors:** Sheng Nie, Mengyi Wang, Qijun Wan, Yaozhong Kong, Jun Ou, Nan Jia, Xiaodong Zhang, Fan Luo, Xiaoting Liu, Lin Wang, Yue Cao, Ruixuan Chen, Mingpeng Zhao, David Yiu Leung Chan, Guobao Wang

**Affiliations:** ^1^State Key Laboratory of Organ Failure Research, National Clinical Research Center for Kidney Disease, Guangdong Provincial Clinical Research Center for Kidney Disease, Nanfang Hospital, Southern Medical University, Guangzhou, China; ^2^Center for Nephrology and Urology Shenzhen University, The First Affiliated Hospital of Shenzhen University, Shenzhen University, Shenzhen, China; ^3^Department of Nephrology, Shenzhen Second People's Hospital, Shenzhen, China; ^4^The First People's Hospital of Foshan, Foshan, China; ^5^Division of Nephrology, Affiliated Hospital of Guilin Medical University, Guilin, China; ^6^Kingmed Diagnostic Laboratory Ltd, Guangzhou, China; ^7^Assisted Reproductive Technology Unit, Department of Obstetrics and Gynaecology, Faculty of Medicine, The Chinese University of Hong Kong, Hong Kong, China

**Keywords:** kidney biopsy, monoclonal gammopathy, monoclonal gammopathy of undetermined significance, monoclonal gammopathy of renal significance, amyloid nephropathy

## Abstract

**Objectives:** To analyze the clinical characteristics and renal pathological manifestations of patients with monoclonal gammopathy (MG) and kidney injury.

**Methods:** This was a multicenter retrospective cohort study conducted at four tertiary hospitals in China. The study population comprised patients with MG admitted from January 1 2013 to December 31 2020. Hospitalization records, laboratory data, and kidney biopsy reports of all patients were collected from the electronic hospital information systems. The study outcomes included kidney disease progression and major hemorrhagic complications after kidney biopsy.

**Results:** We identified 1,164 patients with MG, 782 (67.2%) of whom had underlying kidney injury. Of 101 patients who underwent kidney biopsy, 16 had malignant neoplasms. Amyloid nephropathy was the most common finding (*n* = 34, 33.7%), followed by membranous nephropathy (*n* = 18, 17.8%) and membranoproliferative nephritis (*n* = 8, 7.9%). Among 85 patients with non-malignant hematologic conditions who underwent kidney biopsy, 43 had MG of renal significance (MGRS) related lesions and 42 had MG-unrelated lesions. The risk of kidney disease progression was higher in patients with kidney injury than in patients without kidney injury.

**Conclusion:** Among patients with MG and kidney injury, only 12.9% underwent kidney biopsy and more than 40% of these patients had MG-unrelated lesions. A kidney biopsy is safe and essential to maximize the possibility of correct diagnosis for patients with clinically suspected MG of renal significance (MGRS).

## Introduction

Monoclonal gammopathy (MG) is defined as the presence of a monoclonal immunoglobulin (MIg) in serum resulting from the clonal proliferation of Ig-producing plasma cells or B-lymphocytes (1, 2). Monoclonal gammopathies are a group of disorders ranging from non-malignant small clonal proliferations to malignant neoplasms of plasma cells or B-lymphocytes (3, 4). MG of undetermined significance (MGUS) is usually a premalignant condition, is not associated with any organ damage attributable to the MIg, and does not require treatment (5, 6).

The MIg or its fragments (light chains and/or heavy chains) are secreted into the blood and subsequently filtered by the glomerulus before entering the urine. The kidneys are commonly involved in such hematologic conditions and there are various types of renal disease (7). Kidney damage secondary to MIg in the absence of hematologic malignancy has been increasingly recognized and is called MG of renal significance (MGRS) (8). This term was introduced by the International Kidney and Monoclonal Gammopathy Research Group (IKMG) in 2012 and updated to include all B-cell and plasma cell proliferative disorders that produce a nephrotoxic MIg by the new IKMG consensus in 2019 (9). However, the underlying B-cell or plasma cell clone does not cause tumor complications or meet any current hematologic criteria for specific therapy. Once the hematologic condition progresses to overt multiple myeloma (MM), Waldenström macroglobulinemia (WM), advanced-stage chronic lymphocytic leukemia, or malignant lymphoma (as defined by their respective established disease criteria), these diseases are no longer considered MGRS and affected patients are managed according to disease-specific protocols.

Chronic kidney disease (CKD) is a leading public health problem worldwide. The global estimated prevalence of CKD is 13.4% (11.7–15.1%) (10). During the past decades, the prevalence of CKD and MG has significantly increased, especially among the elderly (11–13). Therefore, it is conceivable that some hospitalized patients with MG also have CKD, but kidney damage is not associated with MIg. Given the different disease management strategies, it is very important to distinguish MGRS from other types of CKD in these patients (14, 15). However, there is uncertainty about which patients should undergo kidney biopsy and when this should be performed among nephrologists and hematologists. Knowledge about clinicopathological features of those patients with MG and kidney injury who needs kidney biopsy is still limited.

In this retrospective cohort study, we described the clinical characteristics of MG patients with kidney injury, the spectrum of kidney biopsy findings in these patients, and the clinical outcomes of these patients. We also evaluated the risk of hemorrhagic complications and the potential benefit of kidney biopsy in these patients.

## Methods

### Study Design, Population, and Data Source

This was a retrospective multicenter cohort study conducted at four tertiary hospitals in China (Nanfang hospital, Guangzhou; Guilin Medical University Affiliated Hospital, Guilin; The First People's Hospital of Foshan, Foshan; The second people's hospital of Shenzhen, Shenzhen). The study cohort comprised patients admitted to a participating hospital from January 1, 2013, to December 31, 2020, who had at least one positive serum immunofixation result during hospitalization. The admission date of the hospitalization during which the patient was first diagnosed with MG was considered the baseline for analysis.

The study centers were asked to export the hospitalization records, laboratory data, and kidney biopsy reports of all patients from the electronic hospital information systems. Hospitalization records consisted of patients' age, sex, date of admission, diagnosis code at admission and discharge, and in-hospital death. Laboratory data included the value and time of patients' serum and urinary tests. Kidney biopsy reports included histologic diagnosis and detailed histologic reports based on light microscopy, electron microscopy, and immunofluorescence assays. The specimens were regularly stained with hematoxylin-eosin, periodic acid Schiff, periodic acid-silver methenamine, and Masson's trichrome, and tested for IgA, IgG, IgM, C3, C4, C1q, and κ/λ light chains by immunofluorescence assays.

The exported data from all study centers were pooled and cleaned at the National Clinical Research Center for Kidney Disease in Guangzhou. The Medical Ethics Committee of Nanfang Hospital approved the study protocol (NFYY-2015-073).

### Identification of MGRS

All patients were divided into two groups (with and without kidney injury) based on their kidney function and urinary test results at baseline for more detailed analysis. Patients were considered to have kidney injury at baseline if they met any of the following inclusion criteria: urinary protein ≥ 1+, estimated glomerular filtration rate (eGFR) ≤ 60 ml/min/1.73 m^2^ (CKD-EPI equation), or daily urinary protein excretion > 0.5 g/24 h.

Among MG patients without hematologic malignancy who underwent kidney biopsy, lesions were considered to be associated with MGRS based on the consensus guideline issued by the IKMG. C3 glomerulopathy and thrombotic microangiopathy are not associated with renal deposition of MIg. Other MGRS-related lesions are caused by deposition of MIgs, fragments thereof, or various aggregation products. Patients with thrombotic microangiopathy were considered to have an MGRS-related lesion if no other obvious cause of thrombotic microangiopathy (such as atypical hemolytic uremic syndrome, thrombotic thrombocytopenic purpura, drugs, or underlying autoimmune disease) was identified.

### Study Outcome

The primary outcome was kidney disease progression, which was defined as progression to end-stage renal disease (sustained eGFR < 15 ml/min/1.73 m^2^ or the need for maintenance renal replacement therapy) or a permanent 30% reduction in the eGFR relative to the initial level at biopsy. The secondary outcome was major hemorrhagic complications, which were defined as hemorrhagic complications requiring blood transfusion or other invasive procedures after kidney biopsy.

### Statistical Analysis

The eGFR was calculated using the CKD-Epidemiology Collaboration equation. SPSS 22.0 statistical software was used. All P values are two-sided. *P* < 0.05 was considered statistically significant. Continuous data were expressed as mean ± standard deviation. Categorical variables were presented as number and percentage. Survival analysis was performed by generating Kaplan-Meier curves and using the log-rank test. Only patients followed up for more than 3 months were included in survival analysis.

## Results

### Study Population

**Figure 1:**
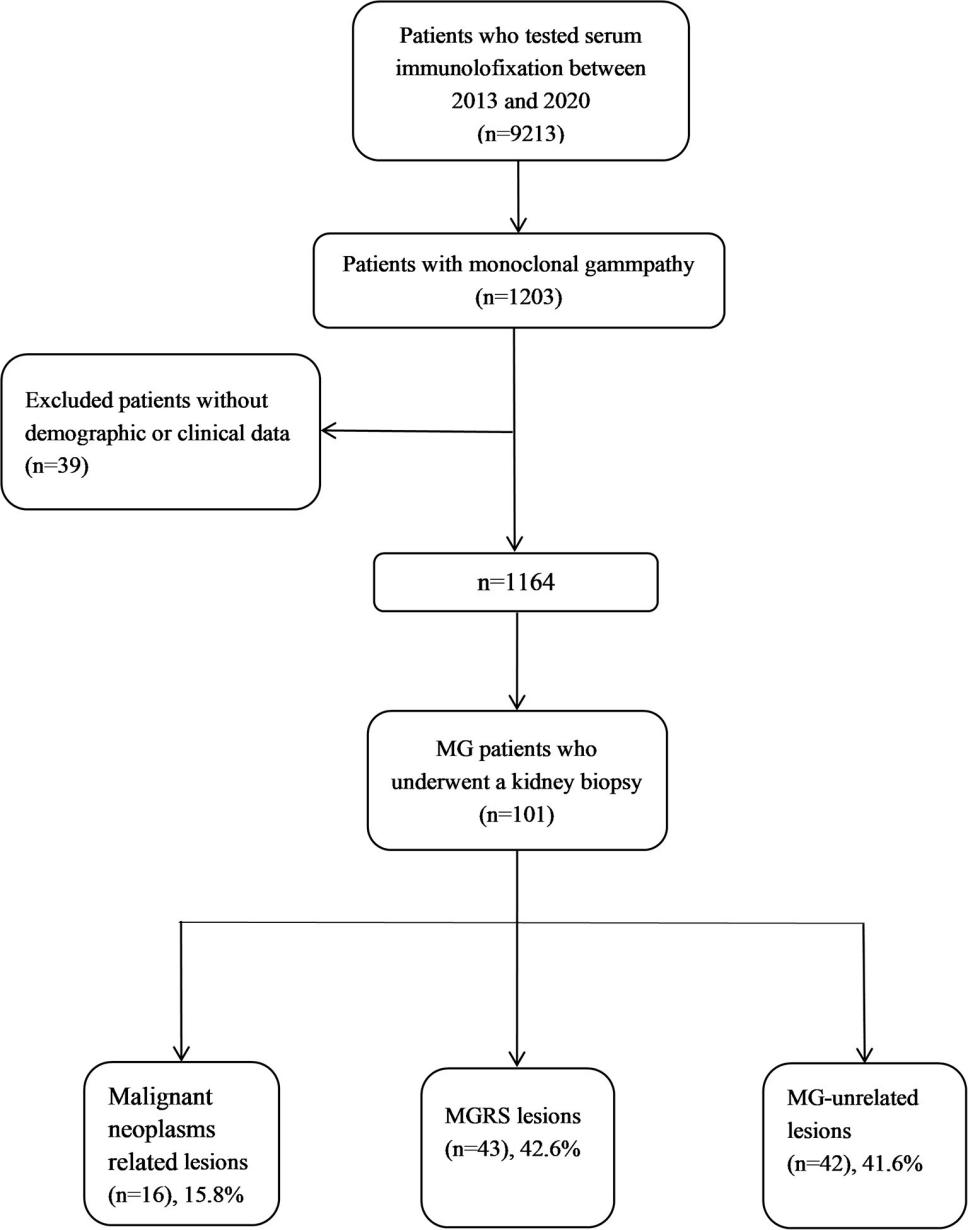
Study flowchart of patients with MG who underwent a kidney biopsy from 2013 to 2020.

Among 9,213 hospitalized patients who underwent a serum immunofixation test during hospitalization, 1,203 had positive results. After excluding 39 patients with missing demographic or clinical data, we identified 1,164 patients who met the inclusion criteria (Figure 1). Of these patients, 782 (67.2%, 782/1,164) had underlying kidney injury, only 101 (12.9%, 101/782) of whom underwent kidney biopsy. The level of serum creatinine, daily excretion of urinary protein, and proportions of patients with hypertension and hematuria were higher among patients with kidney injury than among patients without kidney injury (Table 1). Age, the ratio of serum total light chains (κ to λ), and the proportions of male patients, patients with diabetes, and patients with hepatitis B did not significantly differ between the two groups. The clonal subtype of MIg, including immunoglobulin isotype and immunoglobulin light chain, determined by serum immunofixation is also shown in Table 1. Light chain only was observed with a higher frequency in MG patients with kidney injury (62/782, 7.9% vs. 12/382, 3.1%, MG patients with kidney injury vs. MG patients without kidney injury) and kidney biopsy group (43/101, 42.6 vs. 19/681, 2.8%, kidney biopsy group vs. no kidney biopsy group).

**Table 1:** Baseline characteristics of patients with MG stratified by the status of kidney injury.

**Characteristic**	**Overall (*n* = 1,164)**	**With kidney injury (*n* = 782)**	**Without kidney injury (*n* = 382)**	***P***
Male	711 (61.1%)	481 (61.5%)	230 (60.2%)	0.669
Age (year)	61.2 ± 12.4	62.1 ± 12.3	59.4 ± 12.5	0.001
Hypertension	290 (24.9%)	231 (29.5%)	59 (15.4%)	<0.001
Diabetes	120 (10.3%)	90 (11.5%)	30 (7.9%)	0.054
Hepatitis B	35 (3.0%)	25 (3.2%)	10 (2.6%)	0.587
**Serum studies**				
Albumin (g/l)	31.2 ± 7.4	29.9 ± 7.5	33.8 ± 6.3	<0.001
Creatinine (μmo/l)	166.3 ± 268.2	216.1 ± 315.3	64.3 ± 16.8	<0.001
eGFR (ml/min per 1.73 m^2^)	71.4 ± 36.7	58.5 ± 36.8	97.8 ± 17.1	<0.001
eGFR <60 (ml/min per 1.73 m^2^)	401 (34.5%)	401 (51.3%)	0 (0)	<0.001
κ/λ	1.81 (0.70–7.42)	1.71 (0.70–6.08)	2.10 (0.80–9.81)	0.543
**Urinary studies**				
Urinary protein (g/d)	0.82 (0.20–3.04)	1.44 (0.35–4.15)	0.11 (0.07–0.15)	<0.001
Proteinuria >1.5 g/d	166 (39.6%)	166 (48.0%)	0 (0)	<0.001
Hematuria	407 (38.8%)	407 (56.3%)	0 (0)	<0.001
κ/λ	1.42 (0.49–2.68)	1.42 (0.49–2.68)	/	/
**Hematologic studies**				
Clonal subtype				
Ig isotype				
IgA(+)	305 (26.2%)	212 (27.1%)	93 (24.3%)	0.314
IgG(+)	638 (54.8%)	416 (53.2%)	222 (58.1%)	0.113
IgM(+)	128 (11.0%)	78 (10.0%)	50 (13.1%)	0.111
IgA/IgG/IgM(+)	19 (1.6%)	14 (1.8%)	5 (1.3%)	0.543
IgA&IgG&IgM(−)	74 (6.4%)	62 (7.9%)	12 (3.1%)	0.002
Ig light chain				
Kappa(+)	498 (42.8%)	321 (41.0%)	177 (46.3%)	0.087
Lambda(+)	480 (41.2%)	333 (42.6%)	147 (38.5%)	0.182
Kappa&Lambda(+)	20 (1.7%)	12 (1.5%)	8 (2.1%)	0.49
Kappa&Lambda(−)	166 (14.3%)	116 (14.8%)	50 (13.1%)	0.424
Received BM biopsy	377 (29.0%)	261 (33.4%)	76 (19.9%)	<0.001

*Values for continuous variables are described as mean ± SD or median (IQR) depending on the distribution, and for categoric variables described as count (%)*.

### Clinical Characteristics and Pathological Manifestations of MG Patients Who Underwent Kidney Biopsy

Among patients with MG and kidney injury, 101 underwent kidney biopsy. Table 2 summarizes the demographic and laboratory data of patients who underwent kidney biopsy and those who did not. Patients with kidney injury who underwent kidney biopsy were younger, had higher levels of serum creatinine and daily excretion of urinary protein and had a lower initial eGFR and serum albumin level than patients with kidney injury who did not undergo kidney biopsy.

**Table 2:** Baseline characteristics of MG patients with kidney injury stratified by the status of kidney biopsy.

**Characteristic**	**Kidney biopsy (*n* = 101)**	**Without kidney biopsy (*n* = 681)**	***P***
Male	68 (67.3%)	413 (60.6%)	0.198
Age (year)	56.2 ± 12.0	62.9 ± 12.1	<0.001
Hypertension	36 (35.6%)	195 (28.6%)	0.15
Diabetes	17 (16.8%)	73 (10.7%)	0.072
Hepatitis B	8 (7.9%)	17 (2.5%)	0.004
**Serum studie**s			
Albumin (g/l)	25.8 ± 8.4	30.5 ± 7.2	<0.001
Creatinine (μmo/l)	167.1 ± 182.3	223.3 ± 330.0	0.012
eGFR (ml/min per 1.73 m^2^)	62.7 ± 33.5	57.9 ± 37.3	0.182
eGFR <60 (ml/min per 1.73 m^2^)	46 (45.5%)	355 (52.1%)	0.217
κ/λ	1.35 (0.72–2.12)	1.83 (0.68–9.91)	<0.001
**Urinary studies**			
Urinary protein (g/d)	4.94 (1.67–7.07)	1.10 (0.33–3.27)	<0.001
Proteinuria >1.5 g/d	38 (77.6%)	128 (43.1%)	<0.001
Hematuria	72 (71.3%)	335 (53.9%)	0.001
κ/λ	1.42 (0.49–2.68)	/	/
**Clonal subtype**			
Ig isotype			
IgA(+)	18 (17.8%)	194 (28.5%)	0.024
IgG(+)	30 (29.7%)	386 (56.7%)	<0.001
IgM(+)	10 (9.9%)	68 (10.0%)	0.979
IgA/IgG/IgM(+)	0 (0)	14 (2.1%)	0.146
IgA&IgG&IgM(−)	43 (42.6%)	19 (2.8%)	<0.001
Ig light chain			
Kappa(+)	26 (25.7%)	295 (43.3%)	0.001
Lambda(+)	59 (58.4%)	274 (40.2%)	0.001
Kappa&Lambda(+)	0 (0)	12 (1.8%)	0.179
Kappa&Lambda(−)	16 (15.8%)	100 (14.7%)	0.76
Received BM biopsy	60 (59.4%)	201 (29.5%)	<0.001

*Values for continuous variables are described as mean ± SD or median (IQR) depending on the distribution, and for categoric variables described as count (%)*.

Of 101 patients who underwent kidney biopsy, 16 (15.8%) were diagnosed with malignant neoplasms (15 MM and 1 smoldering MM). Among 85 patients with non-malignant hematologic conditions, 43 had MGRS-related lesions and 42 had MG-unrelated lesions (Table 3). There was no significant difference in the average age, level of serum creatinine and daily excretion of urinary protein, the ratio of serum total light chains (κ to λ) between patients with MGRS-related and MG-unrelated lesions.

**Table 3:** Baseline characteristics of patients with MG with kidney injury who underwent a kidney biopsy.

**Characteristic**	**Kidney biopsy not relevant with malignant neoplasms (*n* = 85)**	**MGRS-related lesions (*n* = 43)**	**MG-unrelated lesions (*n* = 42)**	***P***
Male	60 (70.6%)	26 (60.5%)	34 (81.0%)	0.038
Age (year)	55.8 ± 12.4	57.7 ± 11.0	53.9 ± 13.6	0.161
Hypertension	32 (37.6%)	12 (27.9%)	20 (47.6%)	0.061
Diabetes	15 (17.6%)	4 (9.3%)	11 (26.2%)	0.041
Hepatitis B	7 (8.2%)	3 (7.0%)	4 (9.5%)	0.669
**Serum studies**				
Albumin (g/l)	24.9 ± 8.2	23.6 ± 8.3	26.2 ± 8.0	0.139
Creatinine (μmo/l)	160.1 ± 155.9	166.4 ± 175.3	153.5 ± 135.0	0.705
eGFR (ml/min per 1.73 m^2^)	63.7 ± 33.9	60.0 ± 33.3	67.5 ± 34.5	0.309
eGFR <60 (ml/min per 1.73 m^2^)	39 (45.9%)	24 (55.8%)	15 (35.7%)	0.063
κ/λ	1.35 (0.73–1.91)	0.78 (0.51–1.70)	1.60 (1.26–2.25)	0.57
**Urinary studies**				
Urinary protein (g/d)	5.64 (2.03–7.07)	5.91 (3.57–7.21)	3.37 (1.59–7.96)	0.44
Proteinuria >1.5 g/d	32 (82.1%)	18 (85.7%)	14 (77.8%)	0.52
Hematuria	60 (70.6%)	30 (69.8%)	30 (71.4%)	0.867
κ/λ	1.36 (0.53–2.2)	0.69 (0.21–1.42)	1.98 (1.47–3.77)	0.478
**Hematologic studies**				
Clonal subtype				
Ig isotype				
IgA(+)	15 (17.6%)	7 (16.3%)	8 (19.0%)	0.738
IgG(+)	24 (28.2%)	13 (30.2%)	11 (26.2%)	0.679
IgM(+)	10 (11.8%)	3 (7.0%)	7 (16.7%)	0.166
IgA/IgG/IgM(+)	0 (0)	0 (0)	0 (0)	/
IgA&IgG&IgM(−)	36 (42.4%)	20 (46.5%)	16 (38.1%)	0.432
Ig light chain				
Kappa(+)	21 (24.7%)	7 (16.3%)	14 (33.3%)	0.068
Lambda(+)	51 (60.0%)	29 (67.4%)	22 (52.4%)	0.156
Kappa&Lambda(+)	0 (0)	0 (0)	0 (0)	/
Kappa&Lambda(−)	13 (15.3%)	7 (16.3%)	6 (14.3%)	0.799
Received BM biopsy	49 (57.6%)	27 (62.8%)	22 (52.4%)	0.332

*Values for continuous variables are described as mean ± SD or median (IQR) depending on the distribution, and for categoric variables described as count (%)*.

As shown in Table 4, amyloid nephropathy was the most common MGRS-related lesion, followed by membranoproliferative glomerulonephritis. Eighteen patients were diagnosed with membranous nephropathy, two of whom had MGRS-related lesions and 16 of whom had MG-unrelated lesions. Membranous nephropathy was the most common MG-unrelated lesion. Four patients were diagnosed with diabetic glomerulosclerosis, all of whom had preexisting diabetes mellitus and were considered to have MG-unrelated lesions. The types of the light chain (κ and λ) present were consistent between the serum immunofixation and tissue immunofluorescence assays in 40% of patients. Consistent with the heterogeneity, IgG subclass staining differed across various pathologic manifestions groups in MG patients (Supplementary Table 1).

**Table 4:** Classification of pathological manifestations of MG patients who underwent kidney biopsy.

**Malignant neoplasms related lesions (*N* = 16)**	***n* (%)**	**MGRS-related lesions (*N* = 43)**	***n* (%)**	**MG-unrelated lesions (*N* = 42)**	***n* (%)**
Amyloid nephropathy	8 (50.0%)	Amyloid nephropathy	26 (60.5%)	MN	16 (38.1%)
Glomerular minor lesion	2 (12.5%)	MPGN	4 (9.3%)	Diabetic glomerulosclerosis	4 (9.5%)
FSGS	1 (6.3%)	MN	2 (4.7%)	IgA nephropathy	3 (7.1%)
EPGN	1 (6.3%)	Mesangioproliferative GN	2 (4.7%)	FSGS	3 (7.1%)
MPGN	1 (6.3%)	Mesangial nodular sclerosing glomerulopathy	2 (4.7%)	MPGN	3 (7.1%)
LCDD	1 (6.3%)	Subacute tubulointerstitial nephritis	2 (4.7%)	Glomerular minor lesion	3 (7.1%)
HCDD	1 (6.3%)	C3 GN	1 (2.3%)	Proliferative-sclerosing GN	3 (7.1%)
TMA	1 (6.3%)	EPGN	1 (2.3%)	Subacute tubulointerstitial nephritis	2 (4.8%)
		LCDD	1 (2.3%)	Hypertensive renal disease	1 (2.4%)
		TMA	1 (2.3%)	LN	1 (2.4%)
		Proliferative-sclerosing GN	1 (2.3%)	EPGN	1 (2.4%)
				Mesangioproliferative GN	1 (2.4%)
				Hepatitis B virus-associated nephritis	1 (2.4%)

*FSGS, focal segmental glomurular sclerosis; MN, membranous nephropathy; TMA, thrombotic microangiopathy; MPGN, membranoproliferative glomerulonephritis; LCDD, light chain deposition disease; HCDD, heavy chain deposition disease; EPGN, endocapillary proliferative glomerulonephritis; LN, lupus nephritis*.

### Study Outcomes

**Figure 2:**
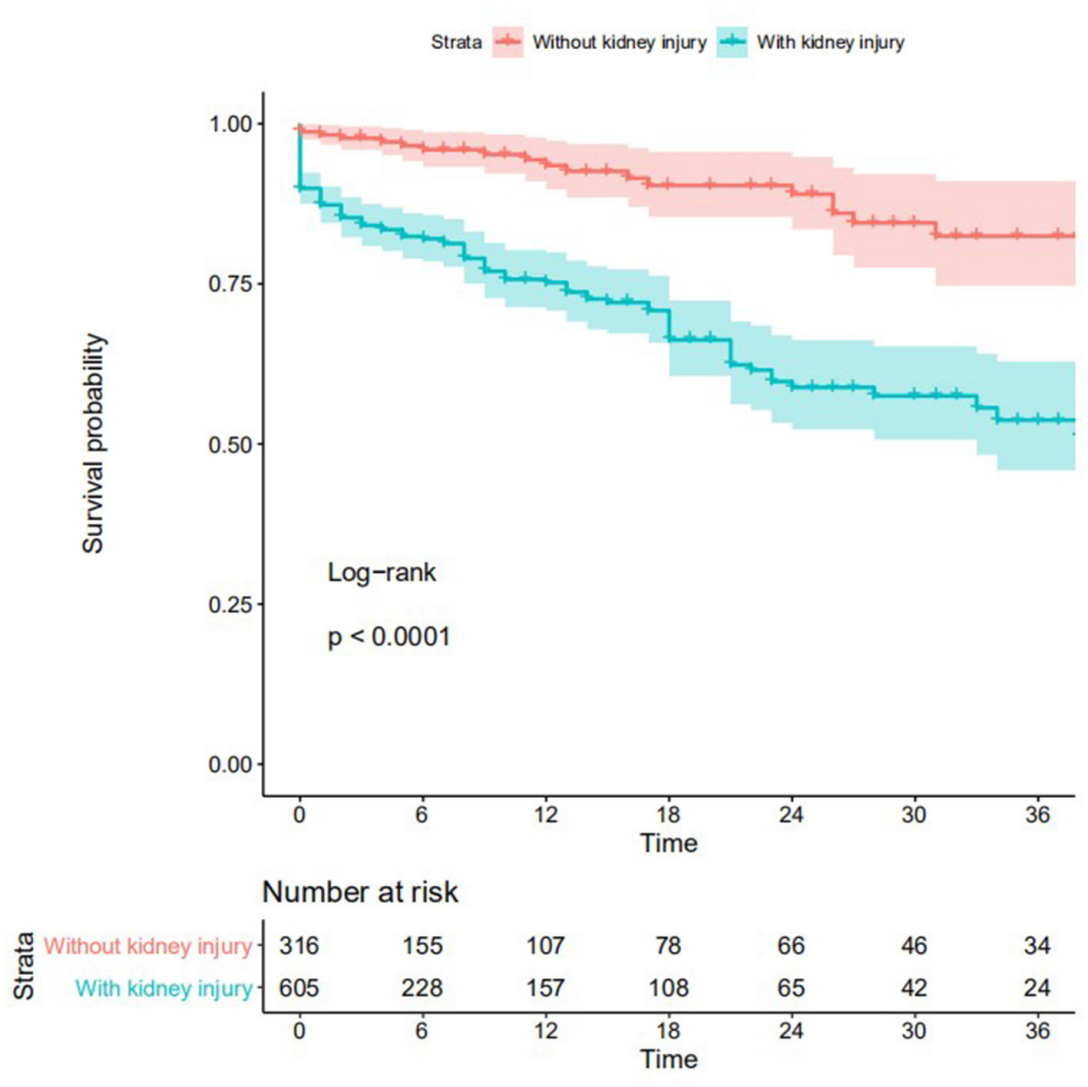
Kaplan–Meier curves of MG patients stratified by the status of kidney injury.

Among 782 patients with MG and kidney injury, 280 (35.8%, 280/782) were followed up for more than 3 months and included in the primary outcome analysis. Over a median of 13 months (interquartile range, 8–22 months) of follow-up, kidney disease progression (the primary outcome) occurred in 58 (20.7%, 58/280) patients. Among 85 non-maligancy MG patients who have received kidney biopsy, kidney disease progression occurred in 1 of 43 (2.3%) patients with MGRS-related lesions and 3 of 42 (7.1%) patients with MG-unrelated lesions. Kaplan-Meier curves in Figure 2 showed that the risk of kidney disease progression was higher in MG patients with kidney injury than in MG patients without kidney injury (*P* < 0.0001). However, the risk of kidney disease progression significantly differ between MG patients who underwent kidney biopsy and those who did not (*P* = 0.03) (Supplementary Figure 1). Of two patients who suffered major hemorrhagic complications after a kidney biopsy, one underwent blood transfusion and the other underwent intra-arterial embolization.

## Discussion

In this multicenter retrospective cohort of 1,164 patients with MG, 782 (67.2%, 782/1,164) had underlying kidney injury. Among them, only 101 (12.9%, 101/782) underwent kidney biopsy and 2 (2.0%, 2/101) suffered from major hemorrhagic complications. Kidney lesions were not related to MG in more than 40% (49.4%, 42/85) of these patients. Kidney biopsy was a safe and simple procedure for pathological diagnosis and prognosis. In our cohort, amyloid nephropathy was found to be the most common MGRS-related lesion, while membranous nephropathy was the most common MG-unrelated lesion. Given that the therapeutic regimens of MGRS and other types of kidney disease are different, our findings suggest that kidney biopsy is essential and can provide clinicians with useful information to guide subsequent treatment.

Over the last decades, the prevalence of CKD and MG has increased dramatically in China. CKD is prevalent in hospitalized patients with MG. Therefore, patients can simultaneously have CKD and MG without a direct causal relationship between the two disorders. The kidney biopsy is the gold standard for diagnosis of most kidney diseases however the procedure was underperformed in our country. There was only 12.9% (101/782) of our study population with kidney biopsy. Except for patients with absolute contraindications (i.e., severe thrombocytopenia), the main reasons why most patients with MG and kidney disease did not undergo kidney biopsy may be that (1) patients with MG and kidney injury were diagnosed with MGRS by default and kidney biopsy was arbitrarily considered unnecessary, and (2) patients refused to undergo kidney biopsy because it is invasive and the risk of hemorrhagic complications might be higher in patients with hematologic disease. Our study showed that kidney biopsy is safe and the risk of massive hemorrhage in MG patients is similar to that reported in general patients (16, 17). More importantly, the major clinical characteristics, such as age, level of serum creatinine and urinary protein, the proportion of hematuria, were not comparable between group with MGRS-related lesions and group with MG-unrelated lesions in our study patients, indicating that the clinical parameters could not be a predictor of finding the MGRS-related lesion. Establishing an MGRS diagnosis hinges on kidney biopsy as it is the only way to demonstrate the presence of monoclonal immunoglobulin deposits in the renal area.

In the IKMG consensus, MGUS does not require treatment, while specific management usually employing chemotherapy toward the pathologic clone and type of kidney injury is recommended for MGRS due to the nephrotoxicity of monoclonal immunoglobulin. MGRS is complex and heterogeneous concerning clinical, pathogenetic, pathologic, and prognostic findings, therefore, a close collaboration between several specialties is required for optimal patient treatment and management. Treatment of MGRS intends to eliminate the underlying clonal plasma cell or B-cell population, and thereby decrease or abolish production of the offending MIg (18). This is the most effective intervention achieved by stem cell transplantation, chemotherapy, and targeted therapeutic regimens developed for MM, acute lymphoblastic leukemia, and B-cell lymphoproliferative disorders (19, 20). According to Ravindran's investigation (3), 13.7% of newly diagnosed MG had chronic kidney diseases, indicating without a kidney biopsy, it is very likely that MG patients with MG-unrelated lesions will be presumably considered as MGRS and treated accordingly. Therefore, clinicians should comprehensively evaluate patients with suspected MGRS, including complete assessment of their renal function and nephrologists should have a low threshold to perform a kidney biopsy.

Consistent with the results of previous studies, amyloid nephropathy was the most common type of MGRS-related lesions in our study, followed by membranoproliferative glomerulonephritis (21). Due to the nephrotoxic potential of MIgs owing to their unique physicochemical properties, the renal disease develops differently in the setting of monoclonal gammopathies (22). Patients with MIg could behave as MGUS present with no sign of renal impairment, however, few studies reported the exact proportion of MG patients with renal impairment. Several small studies showed that kidney injury was observed in 14–58% of patients with specific types of MG while our MG cohort contains 67.2% (782/1,164) renal dysfunction individuals (3, 23, 24). Other patients with MIg show diverse pathological manifestations upon renal biopsy known as MIg-related renal diseases, such as diseases of the glomerulus (amyloid light-chain amyloidosis and MIg deposition disease) and tubules (proximal tubular disorders and cast nephropathy) (25). Most MIg-associated renal diseases develop due to direct deposition of nephrotoxic MIg or its light or heavy chain fragments in various renal tissue compartments. The MIg-related disease is diagnosed based on kidney biopsy and immunofluorescence studies that identify monotypic immunoglobulin deposits (although these are minimal in patients with C3 glomerulopathy and thrombotic microangiopathy).

Furthermore, among patients who underwent kidney biopsy, 18(17.8%, 18/101) and 3(3.3%, 3/101) were diagnosed with membranous nephropathy and IgA nephropathy, respectively. This is unsurprising because these two disorders are the most common type of biopsy-identified glomerulonephritis in general patients (26). Among the 18 patients with membranous nephropathy, 16 (89%, 16/18) with positive PLA2R staining and negative for κ and λ light chains were considered to have MG-unrelated lesions. Meanwhile, the two other patients with negative PLA2R staining and positive for IgG3 and κ or λ light chains were considered to have MG-related membranous nephropathy. Treatment of primary and secondary membranous nephropathy differs. Similarly, treatment of MGRS and primary IgA nephropathy differs. These patients may benefit from accurate pathological diagnosis by kidney biopsy. Although the Kaplan-Meier curves did show a worse prognosis in kidney biopsy group, it may result from selection bias as patients who underwent kidney biopsy had more severe impaired function. Also, long-term follow-up and monitoring are required for some patients who cannot be fully diagnosed after a kidney biopsy. A total of seven MG patients were diagnosed with membranoproliferative glomerulonephritis with high clinical suspicion of MGRS in our study. However, the manifestation of kidney damage (proteinuria/hematuria) in 2 of 7 patients disappeared spontaneously during follow-up, indicating that the kidney lesion was related to other causes (such as infection) rather than to MGRS.

Besides, a higher frequency of light chain only subtype (IgA&IgG&IgM(−)) was observed in MG patients with kidney injury and kidney biopsy group. Light chain excretion, following by deposition and crystallization in kidney, could results in tubular nephropathy. The finding made by Klomjit and coworkers demonstrated a higher concentration of free serum light chain in MG patients with kidney biopsy who tended to have more severe CKD. However, whether the concentration differs among MG subclasses and to what extent it affects the kidney function needs further evacuation.

The investigation conducted in Mayo Clinic depicted that proteinuria ≥1.5 g/d, hematuria and elevated free light chain ratio are potential predictor for MGRS. However, in our cohort, with free light chain ratio inaccessiblem, the clinical measurements, including ratio of κ and λ, proteinuria, hematuria and total light chain showed no differences between patients with MGRS-related lesions and with MG-unrelated lesions. We tend to accounted it to the selection bias in the subcohort since kidney biopsy is more likely to be applied in patients with more severe CKD.

Our study with a large cohort has comprehensively described the clinical characteristics and spectrum of kidney biopsy findings in patients with MG and renal injury. Most previous studies focused on a specific type of MGRS, such as amyloidosis or light chain deposition disease, but without kidney biopsy findings. Furthermore, our cohort is unique because patients with hematologic conditions (MM and WM) were analyzed separately, suggested that we could more accurately describe the characteristics of MGRS and MG-unrelated kidney disease. Finally, this was a multicenter study and the sample size was relatively large. There are some limitations to our study. First, underlying inherent selection bias could not be addressed due to the retrospective nature of this study. Second, important clinical indicators such as free light chain analysis, morphologic assessment, peripheral blood flow cytometry, and myeloma fluorescent *in situ* hybridization were unavailable for most patients. Thus, the correlations between these clinical indicators and MRGS could not be analyzed.

Only 12.9% of MG patients with kidney injury underwent kidney biopsy and more than 40% of these patients had MG-unrelated lesions, among which amyloid nephropathy accounting for the most. Combined the fact that renal insufficiency sigficantly worson the kidney survival in MG patients and the relative low rate of complication after kidney biopsy, we conclude that kidney biopsy is a safe and essential procedure to maximize the possibility of correct diagnosis, and should be encouraged for patients with suspected MGRS.

## Data Availability Statement

The raw data supporting the conclusions of this article will be made available by the authors, without undue reservation.

## Ethics Statement

The studies involving human participants were reviewed and approved by the Medical Ethics Committee of Nanfang Hospital (NFYY-2015-073). Written informed consent to participate in this study was provided by the participants' legal guardian/next of kin.

## Author Contributions

GW and SN contributed to the study design and result interpretation. GW and DC took the lead in drafting the manuscript and received major funding for the study. SN, MW, and QW obtained and analyzed the data. SN, MW, YK, JO, and NJ prepared and cleaned the data. XZ, FL, YC, XL, and RC contributed to the data collection. MZ and DC contributed to revising the manuscript. All authors contributed to the interpretation of data, provided critical revisions to the manuscript, and approved the final draft.

## Conflict of Interest

LW and XL were employed by the company Kingmed Diagnostic Laboratory Ltd, Guangzhou, China. The remaining authors declare that the research was conducted in the absence of any commercial or financial relationships that could be constructed as a potential conflict of interest.
