# Supplementary material for: Kidney Biopsy in Patients With Monoclonal Gammopathy: A Multicenter Retrospective Cohort Study
Source: Front Med (Lausanne). 2021 May 24;8:687149. doi: 10.3389/fmed.2021.687149 (PMC8180861; doi:10.3389/fmed.2021.687149)
Supplement: Supplementary file 1 [file Data_Sheet_1.docx]

**Supplementary Table 1 The proportion of IgG subclass in MG patients with kidney biopsy**

| **Grouping**  **principle** |  | **IgG subclass staining** | **IgG1**  **N (%)** | **IgG2**  **N (%)** | **IgG3**  **N (%)** | **IgG4**  **N (%)** |
| --- | --- | --- | --- | --- | --- | --- |
| **Total** |  | 64.4% 65/101) | 20 (30.7) | 26 (40.0) | 25 (38.4) | 21 (32.3) |
| **MGRS-related or not** | **MGRS-related lesions** | 55.8% (24/43) | 4 (16.6) | 8 (33.3) | 5 (20.8) | 4 (16.6) |
|  | **MG-unrelated lesions** | 69.0% (29/42) | 14 (48.2) | 13 (44.8) | 16 (55.1) | 13 (44.8) |
| **Pathological manifestations classification** | **Amyloid nephropathy** | 67.6% (23/34) | 4 (17.3) | 8 (34.7) | 7 (30.4) | 5 (21.7) |
|  | **MN** | 83.3% (15/18) | 13 (86.6) | 11 (73.3) | 13 (86.6) | 14 (93.3) |
|  | **MPGN** | 50.0% (4/8) | 1 (25.0) | 3 (75.0) | 1 (25.0) | 1 (25.0) |

**Supplementary Figure 1 Kaplan–Meier curves of MG patients stratified by the status of kidney biopsy.**

**
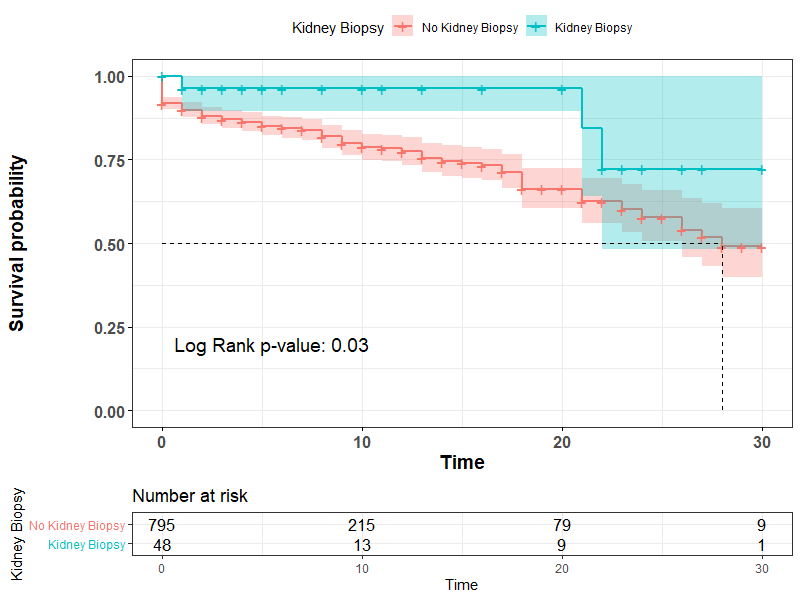
**
